# Comparison of Phenotypic and Functional Characteristics Between Canine Non-B, Non-T Natural Killer Lymphocytes and CD3^+^CD5^dim^CD21^−^ Cytotoxic Large Granular Lymphocytes

**DOI:** 10.3389/fimmu.2018.00841

**Published:** 2018-04-27

**Authors:** Soo-Hyeon Lee, Dong-Jun Shin, Yoseop Kim, Cheol-Jung Kim, Je-Jung Lee, Mee Sun Yoon, Tung Nguyen Thanh Uong, Dohyeon Yu, Ji-Youn Jung, Duck Cho, Bock-Gie Jung, Sang-Ki Kim, Guk-Hyun Suh

**Affiliations:** ^1^Department of Integrated Life Science and Technology, Kongju National University, Yesan-gun, South Korea; ^2^Department of Laboratory and Companion Animal Science, College of Industrial Science, Kongju National University, Yesan-gun, South Korea; ^3^Research Institute for Natural Products, Kongju National University, Yesan-gun, South Korea; ^4^Department of Hemotology-Oncology, Chonnam National University Hwasun Hospital, Hwasun, South Korea; ^5^Department of Radiation Oncology, Chonnam National University Hwasun Hospital, Chonnam National University Medical School, Gwangju, South Korea; ^6^Institute of Animal Medicine, College of Veterinary Medicine, Gyeongsang National University, Jinju, South Korea; ^7^Department of Laboratory Medicine and Genetics, Samsung Medical Center, Sungkyunkwan University School of Medicine, Seoul, South Korea; ^8^Department of Pulmonary Immunology, The University of Texas Health Science Center, Tyler, TX, United States; ^9^Department of Veterinary Internal Medicine, College of Veterinary Medicine, Chonnam National University, Gwangju, South Korea

**Keywords:** natural killer cells, canine, phenotypic modulation, non-B non-T lymphocytes, cytotoxic large granular lymphocytes

## Abstract

Natural killer (NK) cells play a pivotal role in the immune response against infections and malignant transformation, and adopted transfer of NK cells is thought to be a promising therapeutic approach for cancer patients. Previous reports describing the phenotypic features of canine NK cells have produced inconsistent results. Canine NK cells are still defined as non-B and non-T (CD3^−^CD21^−^) large granular lymphocytes. However, a few reports have demonstrated that canine NK cells share the phenotypic characteristics of T lymphocytes, and that CD3^+^CD5^dim^CD21^−^ lymphocytes are putative canine NK cells. Based on our previous reports, we hypothesized that phenotypic modulation could occur between these two populations during activation. In this study, we investigated the phenotypic and functional differences between CD3^+^CD5^dim^CD21^−^ (cytotoxic large granular lymphocytes) and CD3^−^CD5^−^CD21^−^ NK lymphocytes before and after culture of peripheral blood mononuclear cells isolated from normal dogs. The results of this study show that CD3^+^CD5^dim^CD21^−^ lymphocytes can be differentiated into non-B, non-T NK (CD3^−^CD5^−^CD21^−^TCRαβ^−^TCRγδ^−^GranzymeB^+^) lymphocytes through phenotypic modulation in response to cytokine stimulation. *In vitro* studies of purified CD3^+^CD5^dim^CD21^−^ cells showed that CD3^−^CD5^−^CD21^−^ cells are derived from CD3^+^CD5^dim^CD21^−^ cells through phenotypic modulation. CD3^+^CD5^dim^CD21^−^ cells share more NK cell functional characteristics compared with CD3^−^CD5^−^CD21^−^ cells, including the expression of T-box transcription factors (Eomes, T-bet), the production of granzyme B and interferon-γ, and the expression of NK cell-related molecular receptors such as NKG2D and NKp30. In conclusion, the results of this study suggest that CD3^+^CD5^dim^CD21^−^ and CD3^−^CD5^−^CD21^−^ cells both contain a subset of putative NK cells, and the difference between the two populations may be due to the degree of maturation.

## Introduction

Natural killer (NK) cells play a key role in the immune response against infections and malignant transformation through direct cytolytic activity and production of cytokines. There is considerable functional and phenotypic overlap between NK and cytotoxic T cells (1). Unlike cytotoxic T lymphocytes, NK cells exhibit major histocompatibility complex-unrestricted target cell lysis in the absence of prior antigen exposure (2). NK cells also mediate antibody-dependent cellular cytotoxicity, which is one of the most potent mechanisms of NK cell activation through the Fc receptor (3). Recent studies have suggested that adopted transfer of NK cells may be a promising therapeutic approach for cancer patients (4–7). The phenotypic and functional characteristics of NK cells have been documented in various species, including mice, rat, pigs, cows, and humans (8–11). Although the total population of human and mouse NK cells is phenotypically and functionally heterogeneous (12, 13), expression of specific surface molecules, such as CD56 and CD16 on human cells, and NK1.1 and NKp46 on mouse NK cells, allows the identification of NK cells.

In contrast, the phenotypic characteristics of canine NK cells are not completely known. Recently, several researchers suggested NKp46 (NCR1) as a marker of canine NK cells, and CD3^−^CD21^−^ large granular lymphocytes expressing NKp46 are thought to be a population of canine NK cells. However, NKp46 cannot define all NK cells since there is a large subset of circulating NKp46^−^ NK cells that can be induced to express NKp46, and a CD3^+^NKp46^+^ population was also found in peripheral blood mononuclear cells (PBMCs) in healthy dogs (14–16). Therefore, a unique molecule expressed on all canine NK cells that can be used as a specific marker in circulating blood has not yet been identified (17, 18). Previous reports describing the phenotypic features of canine NK cells have produced inconsistent results (18–20). For this reason, canine NK cells are defined as non-B and non-T (CD3^−^CD21^−^) large granular lymphocytes. However, a few reports have demonstrated that canine NK cells share the phenotypic characteristics of T lymphocytes (19–23). The significant discrepancies between reports involving the phenotypic features of putative canine NK cells have prevented the extensive study of these cells.

Previously, we reported that *ex vivo*-expanded canine cytotoxic large granular lymphocytes (CLGLs) exhibiting the morphologic, genetic, and functional characteristics of NK cells expressed both CD3 and CD5^dim^ (17). In addition, in subsequent studies, CD3^−^CD5^−^CD21^−^ NK cells were rapidly expanded following vigorous proliferation of CD3^+^CD5^dim^CD21^−^ cells by prolonging the culture time in modified culture conditions (24–26). Based on these results, the interrelationship between these two cell populations as putative canine NK cells was assumed, and we hypothesized that phenotypic modulation might occur between the two populations during culture in response to activation. In this study, we show that non-B, non-T NK (CD3^−^CD5^−^CD21^−^TCRαβ^−^TCRγδ^−^GranzymeB^+^) lymphocytes can be differentiated from CD3^+^CD5^dim^CD21^−^ cells by cytokine stimulation. The results of this study suggest that a subset of putative canine NK cells is contained in both CD3^+^CD5^dim^CD21^−^ and CD3^−^CD5^−^CD21^−^ cells, and that the difference between the two populations may be the degree of maturation.

## Materials and Methods

### Animals and Blood Collection

Peripheral blood was obtained from 16 healthy beagle dogs (3 years old) which were kept at the animal center at Kongju National University for research or educational purposes. The dogs underwent regular health checkups and blood draws for hematological investigation. All dogs had previously received routine vaccinations against distemper, leptospirosis, parvovirus, and hepatitis, and had been regularly dewormed. Blood collection did not exceed 15 ml/kg of body weight. The use of animals for this study was approved by the Institutional Animal Care and Use Committee of Kongju National University (KNU_2017-02).

### Expansion of NK Cells

Peripheral blood mononuclear cells were isolated by discontinuous density gradient centrifugation with Hypaque-Ficoll gradients (Histopaque 1.119, Sigma-Aldrich, St. Louis, MO, USA; Lymphoprep^TM^ 1.077, Axis-Shield PoC AS, Oslo, Norway). Heparinized whole blood was diluted 1:2 with phosphate-buffered saline (PBS), carefully layered onto the discontinuous density gradients, and centrifuged at 400 × *g* for 25 min. PBMCs were then collected and washed twice with PBS. Canine PBMCs (3.5 × 10^6^) were incubated in a 24-well tissue culture plate with 100-Gy-irradiated-K562 cells (0.5 × 10^6^) in the presence of 100 IU/ml human interleukin (IL)-2 (PeproTec, Rocky Hill, NJ, USA), 10 IU/ml canine IL-15, and 5 ng/ml canine IL-21 (R&D Systems, Minneapolis, MN, USA) in RPMI-1640 and 10% fetal bovine serum (FBS, Gibco, Carlsbad, CA, USA) for 21 days (24). 100-Gy gamma irradiation is a sufficient dose to induce complete K562 cell death regardless of cytokine stimulation (16, 17, 24, 27). Fresh medium with IL-2 and rcIL-15 was provided every other day.

### Flow Cytometry Analysis

Cells were stained as described previously (17). Briefly, fluorescence-activated cell sorting (FACS) analysis was performed using monoclonal antibodies (mAbs) shown in Table 1 according to manufactures instructions. Directly labeled primary antibodies were not available for canine CD11c, CD11d, T cell receptor (TCR) αβ, and TCRγδ, and a sequential staining was performed with fluorescent dye-conjugated secondary antibody (Pacific Blue-conjugated goat anti-mouse IgG) after labeling with unconjugated primary mAbs for these molecules. Expression of Granzyme B, Ki-67, and transcription factors, T-box expressed in T cells (T-bet) and Eomesosermin (Eomes), were measured by intracellular staining using dye-conjugated mAbs shown in Table 1 following cell permeabilization using a Foxp3/Transcription factor staining buffer set (eBioscience, San Diego, CA, USA). Isotype controls were run in parallel. Apoptosis of cells was analyzed using the FITC annexin V/dead cell apoptosis kit (Invitrogen, Carlsbad, CA, USA) according to the manufacturer’s instructions. Flow cytometry analyses were performed using a FACSAria flow cytometer (Becton Dickinson, Franklin Lakes, NJ, USA). Data were analyzed with FlowJo software (Version 10.4.1., FlowJo, LLC, Ashland, OR, USA).

**Table 1:** Antibodies used for flow cytometry in this study.

Antibody	Clone	Conjugates	Supplier
**Primary antibody (Isotype)**			

Mouse anti Human CD14 (IgG2a, κ)*	M5E2	PE-Cy7	BD Pharmingen
Mouse anti-Canine CD21 (IgG1)	CA2.1D6	RPE	Bio-Rad
Mouse anti-Dog CD3 (IgG1)	CA17.2A12	FITC	Bio-Rad
Rat anti-Dog CD5 (IgG2a)	YKIX322.3	APC	Bio-Rad
Rat anti-Dog CD4 (IgG2a)	YKIX302.9	RPE	Bio-Rad
Rat anti-Dog CD8 (IgG1)	YCATE55.9	RPE	Bio-Rad
Mouse anti-Canine CD21 (IgG1)	CA2.1D6	–	Bio-Rad
Mouse anti-Dog CD3 (IgG1)	CA17.2A12	–	Bio-Rad
Mouse anti-Dog CD11c (IgG1)	CA11.6A1	–	Bio-Rad
Mouse anti-Dog CD11d (IgG1)	CA11.8H2	–	Bio-Rad
Canine TCRαβ (IgG1)	CA15.8G7	–	Perter Moore^a^
Canine TCRγδ (IgG2a)	CA20.8H1	–	Perter Moore^a^

**Intracellular staining**			

Mouse anti Human Ki-67 (IgG1, kappa)*	20Raj1	PE-Cyanine7	Invitrogen
Mouse anti Human Granzyme B (IgG1, κ)**	GB11	PE	BD Pharmingen
Mouse anti Human EOMES (IgG1, kappa)^b^	WD1928	PE-Cyanine7	Invitrogen
Mouse anti Human/Mouse T-bet (IgG1, kappa)***	eBio4B10 (4B10)	PE	Invitrogen

**Isotype control**			

Mouse IgG1 Negative Control	–	FITC	Bio-Rad
Mouse IgG1 kappa Isotype Control	P3.6.2.8.1	PE-Cyanine7	Invitrogen
Mouse IgG1 kappa Isotype Control	P3.6.2.8.1	PE	Invitrogen
Mouse IgG1, κ Isotype Control	MOPC-21	PE	BD Pharmingen

**Secondary antibody**			

Goat anti-Mouse IgG (H + L) Secondary Antibody	–	Pacific Blue	Invitrogen

Cross reactivity to the canine equivalent reported* by the supplier.

**In Ref. (15).

***In Ref. (28).

*^a^Leukocyte Antigen Biology Laboratory, University of California, CA, USA*.

*^b^Cross reactivity to dogs was not reported. Amino acid sequences of canine Eomes has 96% identity to its human counterpart*.

### Isolation of CD3^+^CD5^dim^CD21^−^and CD3^−^CD5^−^CD21^−^ Cells

Peripheral blood mononuclear cells or cultured cells were labeled with anti-dog CD3, anti-dog CD5, and anti-dog CD21 fluorescent antibodies as described in Table 1, washed, and suspended in RPMI-1640 media containing 5% FBS. The cells were sorted for two populations, CD3^+^CD5^dim^CD21^−^ and CD3^−^CD5^−^CD21^−^ (non-B, non-T) NK cells, with a FACSAria sorter (Becton Dickinson). CD14^+^ monocytes were not excluded before purification, because it was confirmed that CD14^+^ monocytes were not present in cells expanded for 14 days (Figure S1 in Supplementary Material). The sorted CD3^+^CD5^dim^CD21^−^ and CD3^−^CD5^−^CD21^−^ cell population was greater than 96% pure as determined by FACSAria analysis (Becton Dickinson).

### Real-Time Reverse Transcription-Polymerase Chain Reaction (RT-PCR)

Real-time RT-PCR was performed using a QuantiTect SYBR Green PCR Kit and a Rotor-Gene Q (QIAGEN, Hilden, Germany) according to the manufacturer’s instructions. The cDNA for NK-related genes (CD16, NKG2D, NKp30, NKp44, NKp46, Ly49, perforin, and Granzyme B) were synthesized under the same experimental conditions as reported previously (17). The thermal cycling conditions were 95°C for 15 min followed by 45 cycles at 94°C for 30 s, 53°C for 30 s, and 72°C for 30 s. All samples were tested in triplicate. The relative amount of target gene mRNA was calculated on the basis of its threshold cycle (Ct) compared to the Ct of β-actin. The results are presented as 2^−(Ct of target − Ct of β-actin)^ in arbitrary units.

### Proliferation Assay

Peripheral blood mononuclear cells were adjusted to 1 × 10^6^ cells/ml in PBS and labeled with 5 µM CellTrace™ Violet Cell Proliferation Kit (Thermo Fisher Scientific, MA, USA) for 20 min at 37°C according to the manufacturer’s instructions. After washing twice with PBS, cells were cultured in a 48-well plate at 1 × 10^6^ cells/well in 1 ml media for 7 days. Cells were either stimulated with rcIL-15 (5 ng/ml); canine thyroid adenocarcinoma (CTAC) cells (i.e., canine NK cell sensitive cells, 1 × 10^5^); Convanavalin A (ConA; 1 µg/ml, Sigma-Aldrich); rcIL-15 (5 ng/ml) and rhIL-2 (100 IU/ml, PeproTec); rcIL-15 (5 ng/ml) and CTAC cells (1 × 10^5^); rcIL-15 (5 ng/ml), rhIL-2 (100 IU/ml), and CTAC cells (1 × 10^5^); or medium alone as a negative control. Fresh medium was provided once at day 4. Cells were stained with anti-CD3, anti-CD5, and anti-CD21 fluorescent antibodies after harvest, as described above, and violet fluorescence was evaluated in CD3^+^CD5^bright^CD21^−^, CD3^+^CD5^dim^CD21^−^, and CD3^−^CD5^−^CD21^−^ cells using a FACSAria flow cytometer (Becton Dickinson).

### Enzyme-Linked Immunosorbent Assay (ELISA)

Interferon (IFN)-γ release from purified CD3^+^CD5^dim^CD21^−^ CLGLs and CD3^−^CD5^−^CD21^−^ NK cells in response to CTAC cell incubation was analyzed by ELISA, as previously described (29). Briefly, CTAC cells (5 × 10^4^) were placed in a 96-well microplate in triplicate and cultured at 37°C overnight. The plate was then washed with medium and the purified CLGLs (5 × 10^5^) or NK cells (5 × 10^5^) 12 to 14 days after culture were cocultured with the CTAC cells at a 10:1 E:T ratio without cytokines. After 24 h of coculture, cell-free culture supernatant was harvested and analyzed for IFN-γ production using DuoSet canine IFN-γ kits (R&D Systems, Minneapolis, MN, USA) according to the manufacturer’s instructions. Canine NK-sensitive CTAC cells were used to stimulate NK cells. Cell-free culture supernatant from CTAC and NK cells, and CLGLs, cultured with medium alone for 24 h was used as the control.

### Cytotoxicity Assays

An EZ-Cytox Cell Viability Assay kit (ItsBio, Seoul, Korea) was used to determine the 4 h cytotoxic activity of purified CD3^+^CD5^dim^CD21^−^ CLGLs and CD3^−^CD5^−^CD21^−^ NK cells after culture with CTAC tumor cells, as previously described (29). Briefly, 40,000 CTAC cells were cultured in a 96-well flat-bottom plate in triplicate overnight. The next day, target cells were cultured with purified CD3^+^CD5^dim^CD21^−^ or CD3^−^CD5^−^CD21^−^ cells 12–14 days after culture at a 10:1 effector to target (E:T) ratio at 37°C for 3 h. After adding 10 µl WST-1 (ItsBio) to the well, the plates were incubated for 1 h and placed on ice for 10 min to stop the reaction. Absorption at 450 nm (A_450_) was measured using the Infinite M200 PRO (Salzburg Umgebung, Salzburg, Austria). Percentage of cytotoxicity was calculated using the following equation: 100% − 100 × [A_450_ of effector cell-treated target cells − A_450_ of effector cells (background of effector cells)]/[A_450_ of target cells − A_450_ of target cells with no WST-1 (background of target cells)].

### Statistical Analysis

All statistical analyses were performed using SPSS (Version 24.0, IBM Corp., Armonk, NY, USA). The statistical significance of the differences between three cell subsets was determined using the Kruskal–Wallis test (the nonparametric equivalent of ANOVA) followed by *post hoc* comparison using the Dunn test. The Mann–Whitney *U* test was used for comparisons across two cell populations. The minimal level of significance was *p* < 0.05.

## Results

### Kinetics of Cell Proliferation and Changes in Lymphocyte Subpopulation Composition During PBMC Culture

**Figure 1:**
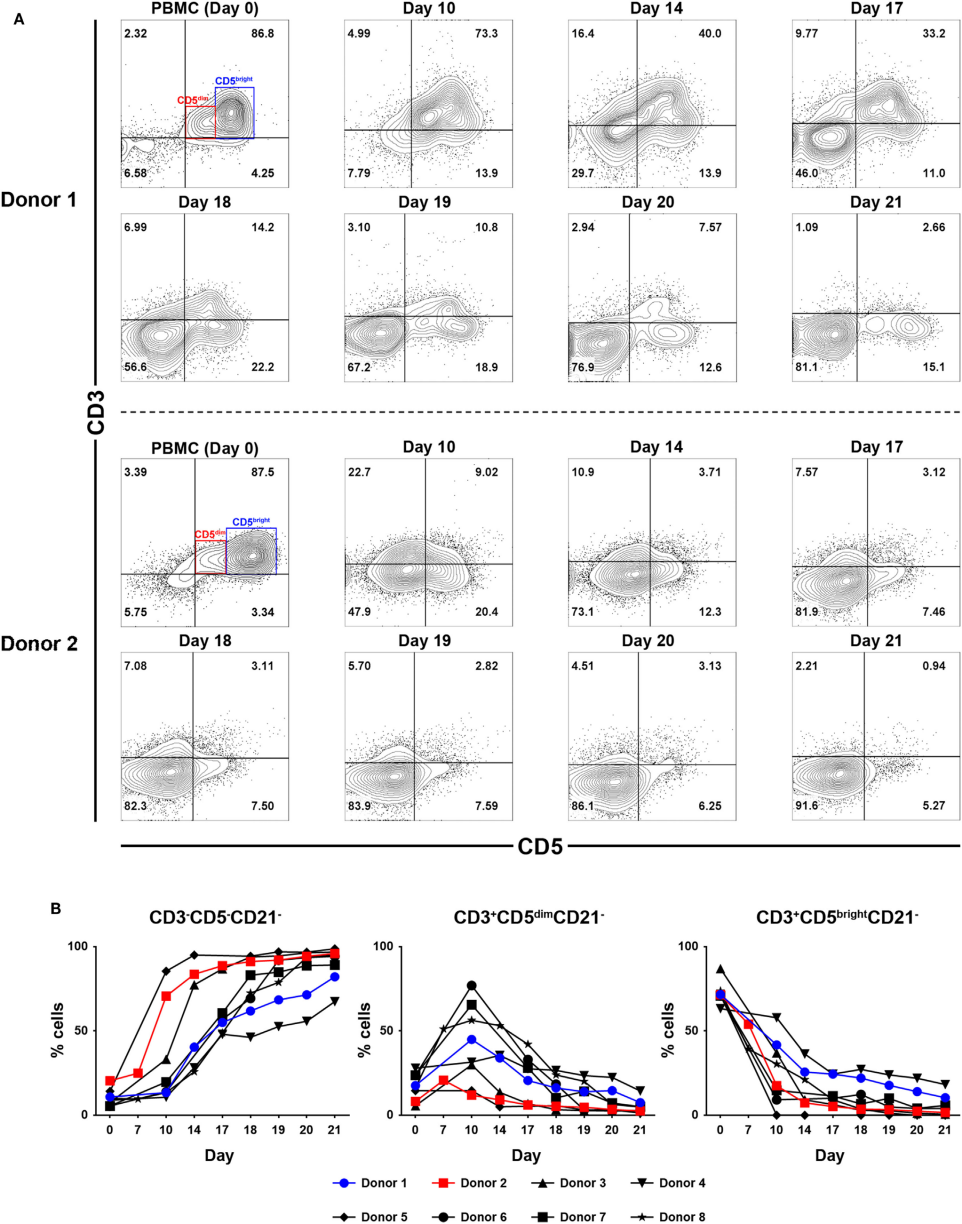
Analysis of lymphocyte subpopulations during *ex vivo* expansion in the presence of 100-Gy-irradiated K562 cells, interleukin (IL)-2, IL-15, and IL-21. **(A)** Representative flow cytometry data (*n* = 8), and **(B)** lymphocyte subpopulation dynamics showing changes in the frequency of CD3^−^CD5^−^CD21^−^, CD3^+^CD5^dim^CD21^−^, and CD3^+^CD5^bright^CD21^−^ cell populations during proliferation in culture of peripheral blood mononuclear cells (PBMCs) for 21 days. The results are shown as the mean ± SD measured from eight different donors.

**Figure 2:**
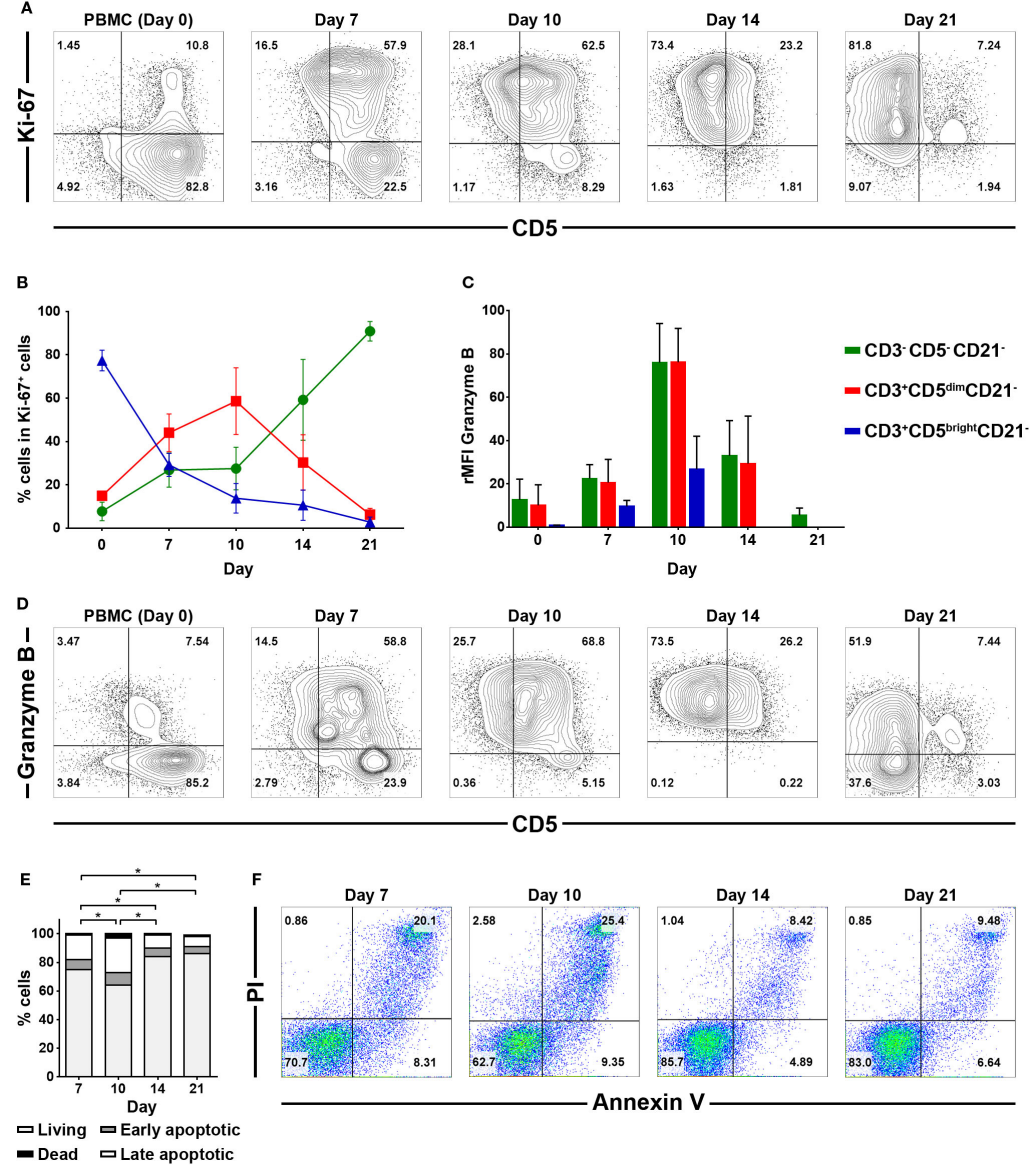
Intracellular expression of Ki-67 and Granzyme B in CD3^−^CD5^−^CD21^−^, CD3^+^CD5^dim^CD21^−^, and CD3^+^CD5^bright^CD21^−^ cell compartments, and analysis of cell death frequency during PBMC culture for 21 days. **(A)** Flow cytometric analysis of Ki-67 expression (*n* = 9). **(B)** Changes in the frequency of each cell population within Ki-67-expressing cells during culture for 21 days (*n* = 9). **(C)** Changes in the frequency of Granzyme B intracellular expression in CD3^−^CD5^−^CD21^−^, CD3^+^CD5^dim^CD21^−^, and CD3^+^CD5^bright^CD21^−^ cell populations during PBMC culture for 21 days. Expression level of Granzyme B represents relative mean fluorescence intensity (rMFI) (*n* = 9). **(D)** Representative flow cytometry data of the frequency of Granzyme B intracellular expression in each cell population during culture for 21 days (*n* = 9). **(E)** The frequency of cell death during culture for 21 days. Whole cells were stained with annexin V/propidium iodide (PI) on days 7, 10, 14, and 21 of culture. The population of cells that were negative for both annexin V and PI was defined as living cells. Annexin V^+^/PI^−^ cells were classified as early apoptotic cells, and double-positive cells were classified as late apoptotic cells. Annexin V^−^/PI^+^ cells were classified as dead cells (*n* = 5). **(F)** Representative flow cytometry data of annexin V/PI staining of cells during culture to evaluate the frequency of cell death frequency among the total cell population (*n* = 5).

To determine which cell populations proliferate predominantly during culture, changes in the proportion of CD3^+^CD5^bright^CD21^−^, CD3^+^CD5^dim^CD21^−^ and CD3^−^CD5^−^CD21^−^ lymphocytes in culture were evaluated. Serial phenotypic analysis identified a rapid expansion of CD3^+^CD5^dim^CD21^−^ and CD3^−^CD5^dim^CD21^−^ lymphocytes, which became dominant in the culture. Significantly reduced numbers of CD3^+^CD5^bright^CD21^−^ lymphocytes were found starting on day 7 after culture. This was followed by a striking shift in CD3^−^CD5^−^CD21^−^ subsets, with a dramatic decrease in the CD3^+^CD5^dim^CD21^−^ cell fraction from days 14 to 17 of culture, depending on the donor. Most of the cells in culture were CD3^−^CD5^−^CD21^−^ at day 21 (Figures 1A,B; gating strategy in Figure S2 in Supplementary Material). To determine whether the increased numbers of CD3^−^CD5^−^CD21^−^ cells were a consequence of the rapid cell proliferation, or alternatively, phenotypic switching of CD3^+^CD5^dim^CD21^−^ cells, expression of intracellular Ki-67, an indicator of cell proliferation, was analyzed within the lymphocyte subsets by flow cytometry at days 0, 7, 10, 14, and 21. As shown in Figures 2A,B, compared with CD3^+^CD5^bright^CD21^−^ and CD3^−^CD5^−^CD21^−^ cells, in which Ki-67 expression was low, up to 78% of CD3^+^CD5^dim^CD21^−^ cells expressed Ki-67 during the first 7–10 days of culture. Interestingly, the phenotype of Ki-67-expressing cells gradually moved from CD3^+^CD5^dim^CD21^−^ to CD3^−^CD5^−^CD21^−^, and a rapid increase in the frequency of Ki-67-expressing CD3^−^CD5^−^CD21^−^ cells was observed around day 14 of culture (Figures 2A,B). The proportion of CD3^+^CD5^dim^CD21^−^ cells abruptly declined by day 21 (Figures 1 and 2). Most Ki-67-expressing cells simultaneously expressed Granzyme B, and the expression of Granzyme B decreased in cultured cells at day 21 (Figures 2C,D). The percentage of cells undergoing death during culture was determined by the expression of propidium iodide (PI) and annexin V in the total cell population. The frequency of annexin V^+^ and PI^+^ apoptotic/necrotic cells at days 7 and 10 of culture was 16.9 ± 2.4 and 24.4 ± 1.6%, respectively. Moreover, the frequency of apoptotic/necrotic cells was significantly decreased after 14 (8.9 ± 0.9%) and 21 days (7.4 ± 1.3%) of culture (*p* < 0.05) (Figures 2E,F).

### Phenotypic Changes in Isolated CD3^+^CD5^dim^CD21^−^ Cells During Culture

**Figure 3:**
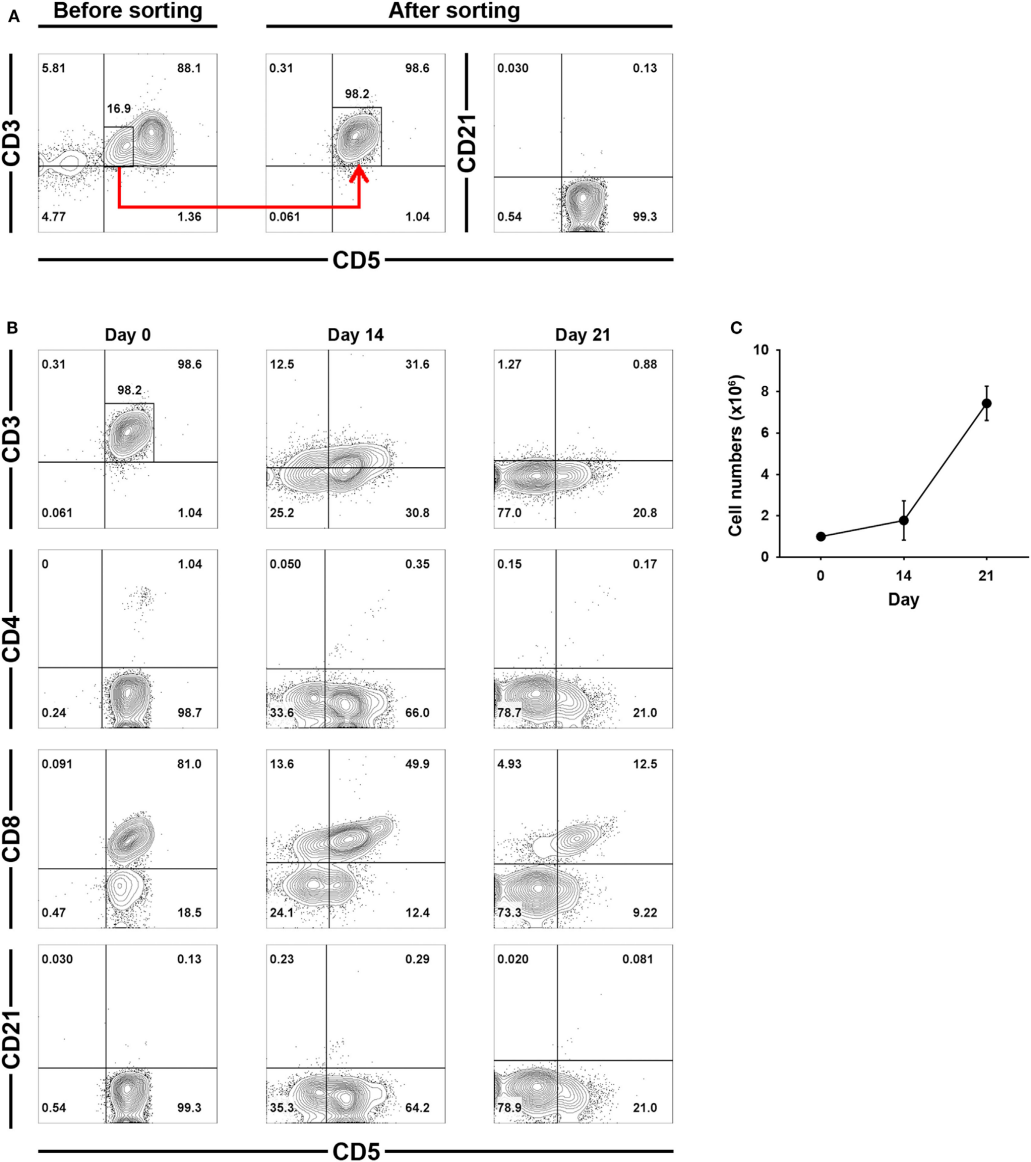
Phenotypic changes of purified CD3^+^CD5^dim^CD21^−^ cells during culture for 21 days. **(A)** CD3^+^CD5^dim^CD21^−^ cells were purified from freshly isolated PBMCs using a cell sorter. The purity of CD3^+^CD5^dim^CD21^−^ cells after sorting was more than 98% (*n* = 5). **(B)** Phenotypic analysis of the purified CD3^+^CD5^dim^CD21^−^ cells during culture for 21 days. CD3^−^CD5^−^CD21^−^ cells were proliferated from purified CD3^+^CD5^dim^CD21^−^ cells, and the majority of expanded cells in culture were CD3^−^CD5^−^CD21^−^ after 21 days. The phenotype of most of these expanded CD3^−^CD5^−^CD21^−^ cells was CD4^−^ CD8^+/−^ after 21 days of stimulation. The results shown are representative results from one of five different donors. **(C)** The rate of proliferation was calculated by counting the total number of viable cells in culture. Values represent the mean ± SD (*n* = 5).

To confirm that the increased numbers of CD3^−^CD5^−^CD21^−^ cells during culture was a direct consequence of CD3^+^CD5^dim^CD21^−^ cell phenotypic modulation, CD3^+^CD5^dim^CD21^−^ cells were purified from PBMCs (98 ± 1.5% purity) using flow cytometry (Figure 3A), and were cultured for 21 days. The non-B, non-T (CD3^−^CD5^−^CD21^−^) cells expanded from the CD3^+^CD5^dim^CD21^−^ cells (Figures 3B,C). The number of CD3^−^CD5^dim^CD21^−^ and CD3^−^CD5^−^CD21^−^ cells gradually increased in culture, while the frequency of CD3^+^CD5^dim^CD21^−^ cells decreased during expansion. After 21 days of culture, most cells were CD3^−^CD5^−^CD21^−^ (80.8 ± 3.7%). A few CD3^−^CD5^dim^CD21^−^ lymphocytes (18.4 ± 2.2%) were present, but all CD3^+^CD5^dim^CD21^−^ cells disappeared (Figure 3B). Expression patterns of TCRαβ and TCRγδ also changed markedly in the process of phenotypic modulation of CD3^+^CD5^dim^CD21^−^ cells to CD3^−^CD5^−^CD21^−^ cells during culture, and most of the expanded CD3^−^CD5^−^CD21^−^ cells (0.99 ± 0.90%) did not express TCRαβ or TCRγδ after 21 days of culture (Figures S3 and S4 in Supplementary Material). The phenotype of most of the expanded CD3^−^CD5^−^CD21^−^ cells was CD4^−^ CD8^+^^/−^ TCRαβ^−^ TCRγδ^−^ CD11c^+/−^ CD11d^−^ after 21 days of stimulation (Figure 3B; Figure S3 in Supplementary Material).

### mRNA Expression of NK Cell-Associated Genes in Each Subset of Lymphocytes

**Figure 4:**
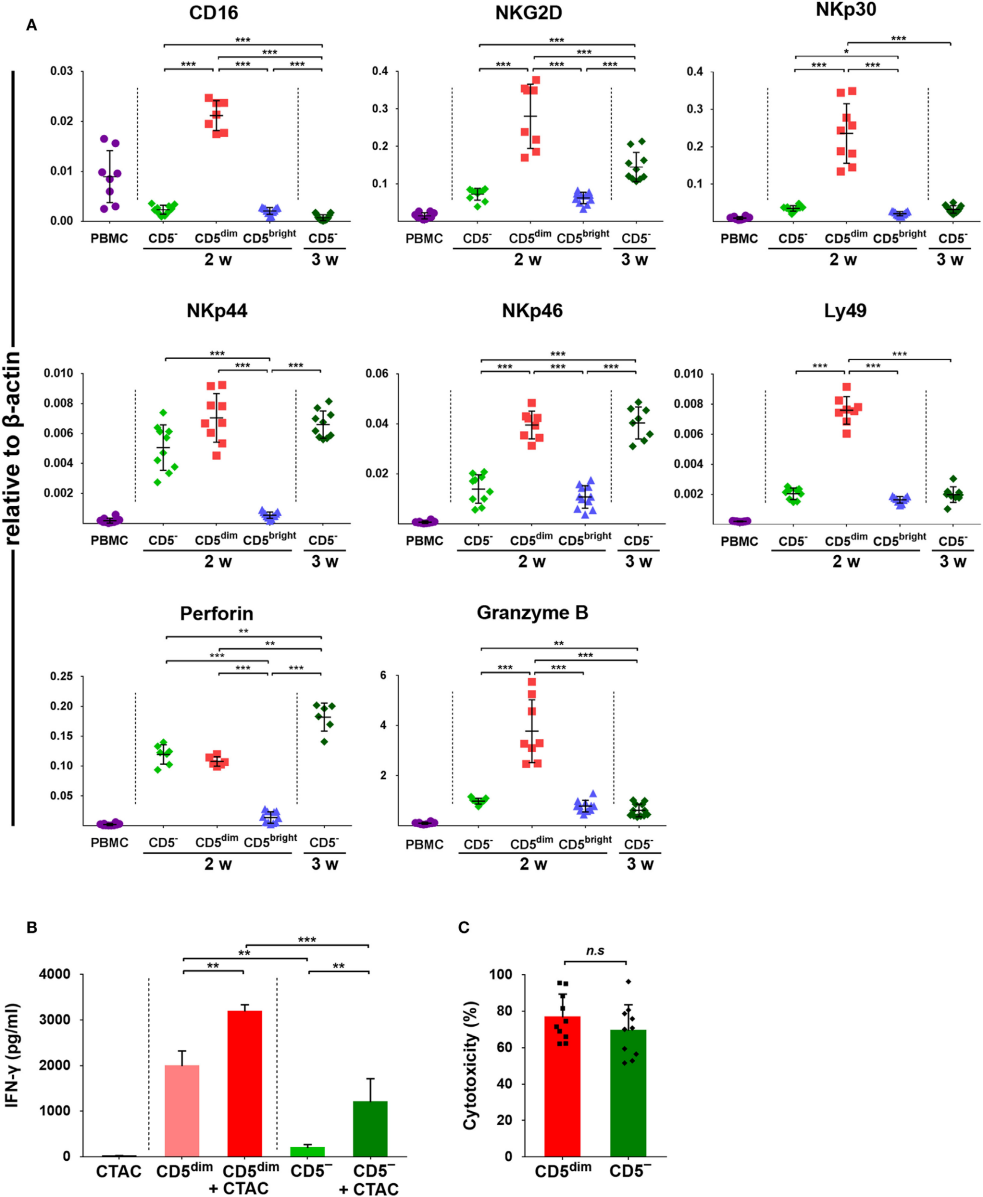
Expression of NK-associated receptor and function of purified CD3^+^CD5^dim^CD21^−^ and CD3^−^CD5^−^CD21^−^ lymphocytes. **(A)** mRNA expression of NK cell-related molecules including CD16, NKG2D, NKp30, NKp44, NKp46, perforin, and Granzyme B in canine PBMCs and purified CD3^+^CD5^dim^CD21^−^ (CD5^dim^), CD3^+^CD5^bright^CD21^−^ (CD5^bright^), and CD3^−^CD5^−^CD21^−^ (CD5^−^) lymphocytes 2 (2w) or 3 weeks (3w) after expansion (*n* = 11). Levels were determined by real-time reverse transcription-polymerase chain reaction. The mRNA levels were expressed relative to β-actin. **(B)** Production of interferon (IFN)-γ in purified CD3^+^CD5^dim^CD21^−^ and CD3^−^CD5^−^CD21^−^ lymphocytes (*n* = 10). IFN-γ production was assessed in the culture supernatants by enzyme-linked immunosorbent assay. Control conditions consisted of the culture supernatant from canine thyroid adenocarcinoma (CTAC) cells, CD3^+^CD5^dim^CD21^−^ (CD5^dim^) or CD3^−^CD5^−^CD21^−^ cells (CD5^−^) cultured alone for 24-h without cytokines. IFN-γ production levels in each subset of lymphocytes are shown as the mean ± SD. *p < 0.01, **p < 0.005, and ***p < 0.001. **(C)** The natural cytotoxicity against CTAC cells of purified CD3^+^CD5^dim^CD21^−^ (CD5^dim^) and CD3^−^CD5^−^CD21^−^ (CD5^−^) cells 2 weeks after culture. The 4-h cytotoxicity was measured at 10:1 effector to target ratio. The results are shown as mean ± SD percentages of cytotoxicity (*n* = 5, each tested in duplicate).

Expression of CD16, NKG2D, NKp30, NKp44, NKp46, perforin, Granzyme B, and Ly49, was investigated in FACS-sorted lymphocyte subsets around 14 and 21 days after culture using quantitative RT-PCR due to the lack of species-specific mAbs. As shown in Figure 4A, the mRNA levels of NK-related genes in CD3^+^CD5^dim^CD21^−^ cells purified after around 2 weeks (12 to 14 days) of culture were significantly upregulated compared to CD3^+^CD5^bright^CD21^−^ cells purified at the same time and freshly isolated PBMCs (*p* < 0.001). With the exception of NKp44 and perforin, which were not statistically different between cell subsets, mRNA levels were also upregulated compared to CD3^−^CD5^−^CD21^−^ cells isolated around 2 weeks after culture (*p* < 0.001). mRNA levels of NKG2D, NKp46, and perforin in CD3^−^CD5^−^CD21^−^ cells purified at 3 weeks (21 days) of culture were significantly increased compared with cells purified after 2 weeks of culture (*p* < 0.001). mRNA level of perforin in CD3^−^CD5^−^CD21^−^ cells purified at 3 weeks was significantly upregulated compared to that in CD3^+^CD5^dim^CD21^−^ cells purified after 2 weeks of culture (*p* < 0.005).

### IFN-γ Production and Cytotoxic Activities of CD3^+^CD5^dim^CD21^−^ and CD3^−^CD5^−^CD21^−^ Cells Against Tumor Cells

To compare the functional characteristics between expanded CD3^+^CD5^dim^CD21^−^ and CD3^−^CD5^−^CD21^−^ cells, the IFN-γ production and natural cytolytic activities of each cell population against canine NK-sensitive CTAC cells were analyzed. The levels of IFN-γ were evaluated in the culture supernatants derived from the purified CD3^+^CD5^dim^CD21^−^ or CD3^−^CD5^−^CD21^−^ cells after a 24-h stimulation with or without CTAC cells. As shown in Figure 4B, CD3^+^CD5^dim^CD21^−^ cells exhibited significantly higher IFN-γ production compared to CD3^−^CD5^−^CD21^−^ cells stimulated with or without CTAC cells (*p* < 0.05). The concentration of IFN-γ was markedly elevated when CD3^+^CD5^dim^CD21^−^ or CD3^−^CD5^−^CD21^−^ cells were cocultured with CTAC cells compared to when each subset of lymphocytes was cultured in the absence of tumor cells (*p* < 0.05). Next, the cytotoxicity against CTAC cells of purified CD3^+^CD5^dim^CD21^−^ and CD3^−^CD5^−^CD21^−^ cells after culture were measured at 10:1 effector to target ratio. As shown in Figure 4C, no significant differences were found in cytotoxicity between CD3^+^CD5^dim^CD21^−^ and CD3^−^CD5^−^CD21^−^ cells. The mean cytotoxicity of CD3^+^CD5^dim^CD21^−^ and CD3^−^CD5^−^CD21^−^ cells was 76.6 ± 12.8 and 69.3 ± 14.3%, respectively.

### Expression of Eomes and T-bet Transcription Factors in Lymphocyte Subsets Before and After Culture

**Figure 5:**
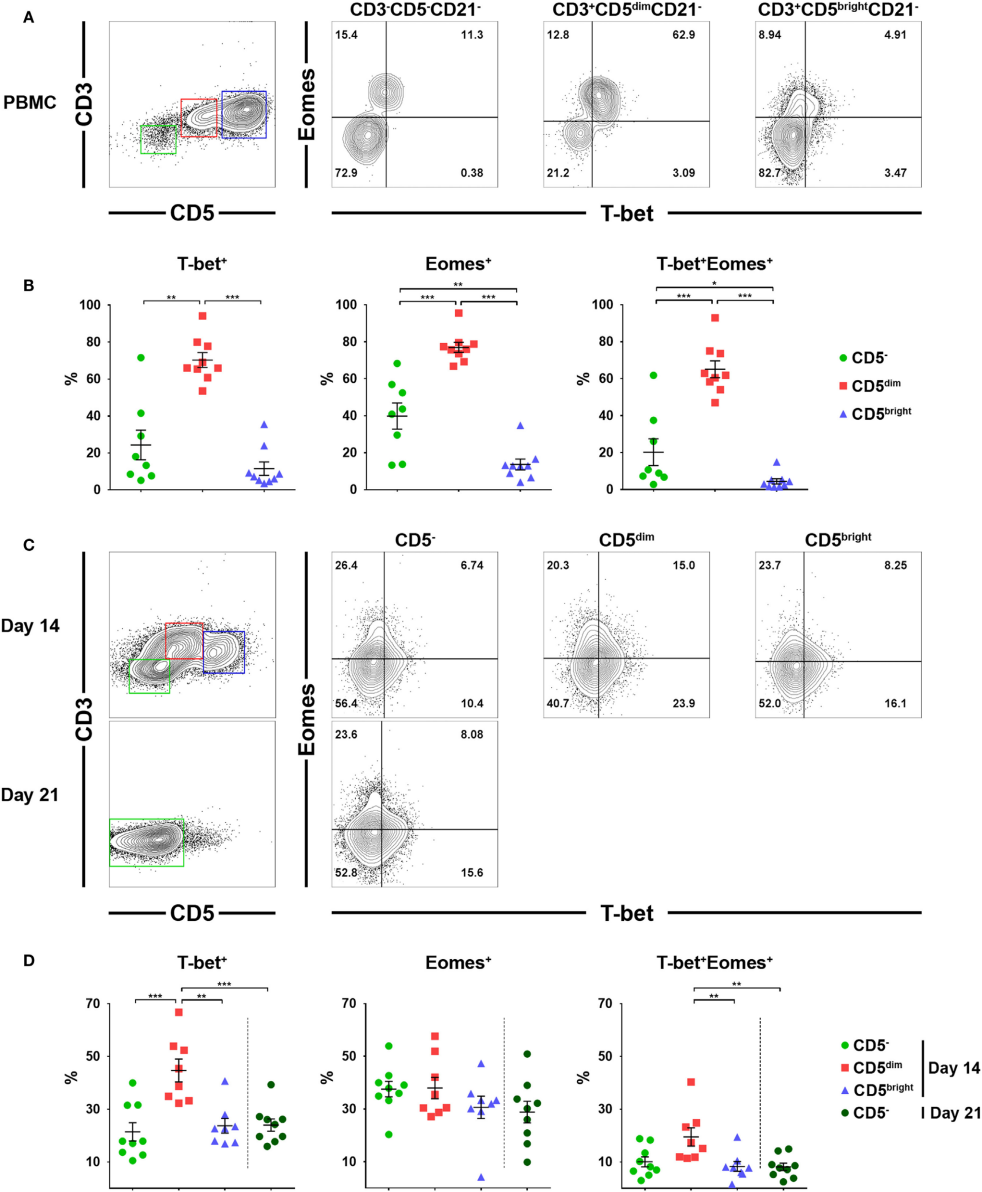
Expression of the T-box transcription factors, T-box expressed in T cells (T-bet) and Eomesosermin (Eomes) in each lymphocyte subset in relation to the expression of CD3, CD5, and CD21 before and after culture of PBMCs. **(A)** Expression of T-bet and Eomes in CD3^+^CD5^dim^CD21^−^ (red), CD3^+^CD5^bright^CD21^−^ (blue), and CD3^−^CD5^−^CD21^−^ (green) lymphocytes before culture. The results shown represent those obtained from one of nine different donors. **(B)** The percentage of cells expressing T-bet, Eomes, and both factors in CD3^+^CD5^dim^CD21^−^ (CD5^dim^), CD3^+^CD5^bright^CD21^−^ (CD5^bright^), and CD3^−^CD5^−^CD21^−^ (CD5^−^) lymphocytes before culture (*n* = 9). **(C)** Expression of T-bet and Eomes in CD3^+^CD5^dim^CD21^−^ (CD5^dim^, red), CD3^+^CD5^bright^CD21^−^ (CD5^bright^, blue), and CD3^−^CD5^−^CD21^−^ (CD5^−^, green) lymphocytes 2 weeks (2w) after culture, and in CD3^−^CD5^−^CD21^−^ cells 3 weeks (3w) after culture. The results shown represent those obtained from one of nine different donors. **(D)** The percentage of cells expressing T-bet, Eomes, and both factors in CD3^+^CD5^dim^CD21^−^ (CD5^dim^), CD3^+^CD5^bright^CD21^−^ (CD5^bright^), and CD3^−^CD5^−^CD21^−^ (CD5^−^) lymphocytes 2 (2w) or 3 weeks (3w) after culture (*n* = 9) (*p < 0.01, **p < 0.005, and ***p < 0.001).

Previous studies have suggested that T-bet and Eomes are the key transcription factors that regulate the development, peripheral maturation, and function of NK cells. In addition, mature NK cells express high levels of these factors in mice and humans (30, 31). Therefore, we evaluated the expression of T-bet and Eomes in each lymphocyte subset before and after culture using flow cytometry. Gates were set according to fluorescence minus one controls (Figure S5 in Supplementary Material). As shown in Figures 5A,B, a significantly higher frequency of CD3^+^CD5^dim^CD21^−^ cells expressed T-bet (70.3 ± 12.0%) and Eomes (77.0 ± 8.2%) compared to CD3^−^CD5^−^CD21^−^ (T-bet, 24.4 ± 22.7%; Eomes, 39.8 ± 19.9%) or CD3^+^CD5^bright^CD21^−^ (T-bet, 11.5 ± 10.9%; Eomes, 13.6 ± 8.8%) cells before culture (*p* < 0.001). Approximately 65% of CD3^+^CD5^dim^CD21^−^ cells before culture coexpressed T-bet and Eomes, whereas a small number of CD3^−^CD5^−^CD21^−^ cells (20.2 ± 20.5%) and very few CD3^+^CD5^bright^CD21^−^ cells (4.4 ± 4.2%) coexpressed these factors. The frequency of cells expressing T-bet and Eomes in CD3^+^CD5^dim^CD21^−^ cells significantly decreased with time (*p* < 0.05), while the expression of the two transcription factors in CD3^−^CD5^−^CD21^−^ cells was not different after 2 or 3 weeks of culture (Figures 5C,D). The frequency of T-bet expression in CD3^+^CD5^dim^CD21^−^ cells (44.7 ± 12.3%) was still higher compared to CD3^−^CD5^−^CD21^−^ (21.4 ± 10.3%) and CD3^+^CD5^bright^CD21^−^ (23.7 ± 7.8%) cells 2 weeks after culture (*p* < 0.001). However, the frequency of Eomes expression in CD3^+^CD5^dim^CD21^−^ cells (38.0 ± 11.5%) decreased to the same level as CD3^−^CD5^−^CD21^−^ (37.6 ± 8.8%) and CD3^+^CD5^bright^CD21^−^ (30.7 ± 12.1%) cells 2 weeks after culture (Figures 5C,D). The number of T-bet and Eomes double-positive CD3^+^CD5^dim^CD21^−^ cells (19.5 ± 9.9%) also decreased to levels similar to CD3^−^CD5^−^CD21^−^ cells (10.0 ± 5.7%) after 2 weeks of culture (Figures 5C,D). The expression of T-bet (24.0 ± 7.0%) and Eomes (28.9 ± 12.3%) in CD3^−^CD5^−^CD21^−^ cells was no different after 3 weeks of culture compared to the same population after 2 weeks of culture (Figures 5C,D).

### Proliferative Capacity of Each Lymphocyte Subset in Response to Stimulation With Cytokines or Canine NK Cell-Sensitive Tumor Cells

**Figure 6:**
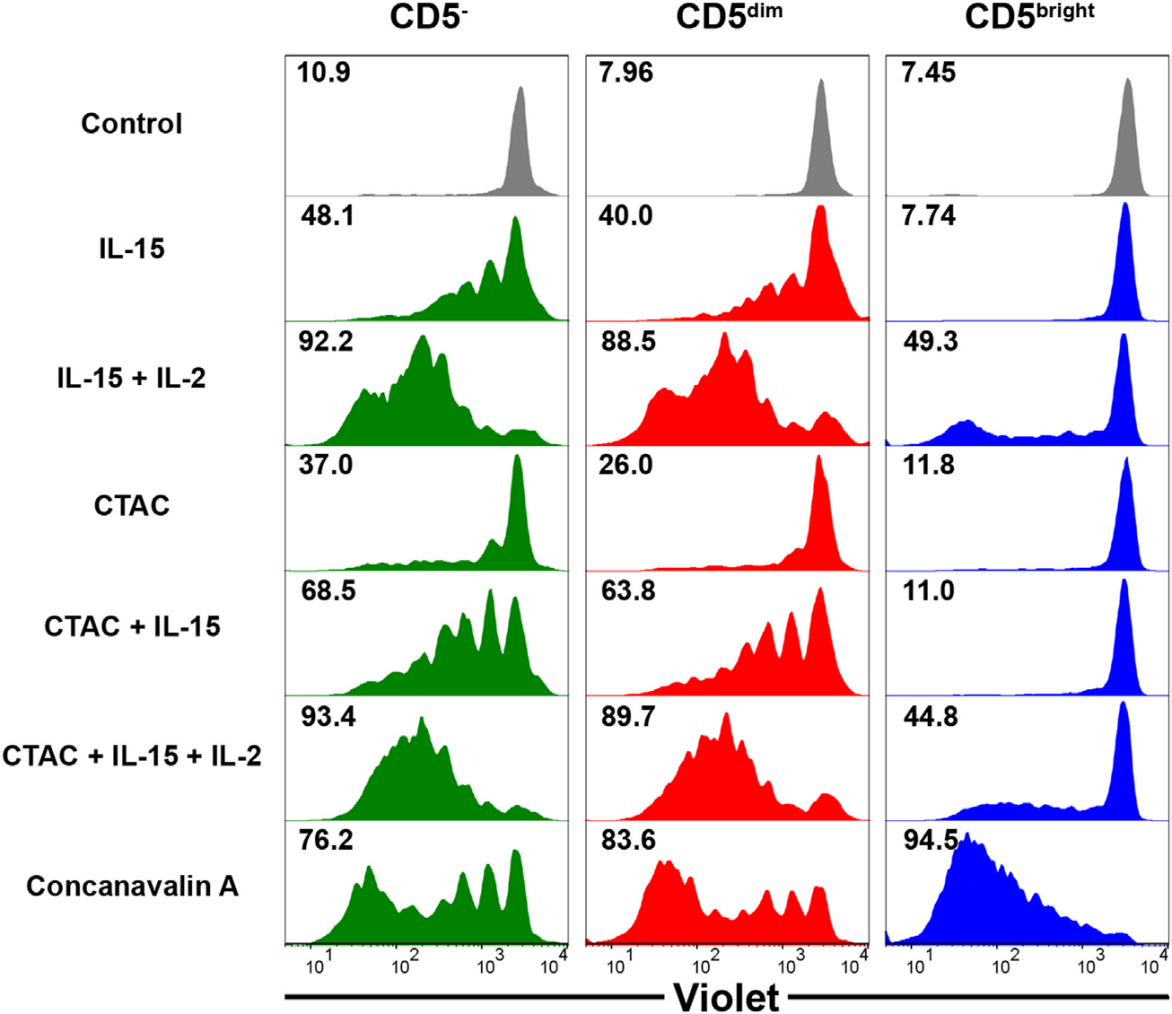
Comparison of the proliferative capacity in CD3^−^CD5^−^CD21^−^ non-B, non-T (CD5^−^), CD3^+^CD5^dim^CD21^−^ (CD5^dim^), and CD3^+^CD5^bright^CD21^−^ (CD5^bright^) lymphocytes. PBMCs were stained with Violet Cell Trace Dye before culture. To evaluate the proliferation of the different lymphocyte subsets, cells were stimulated with IL-15; IL-2; and IL-15; canine NK cell-sensitive canine thyroid adenocarcinoma (CTAC) cells; CTAC cells and IL-15; CTAC cells, IL-2, and IL-15; or concanavalin A for 7 days. Cells cultured in medium alone served as a negative control. Results are representative of data from five different donors. The percentage of proliferating cells within the respective subsets is indicated.

To compare the proliferative capacity of CD3^+^CD5^dim^CD21^−^, CD3^+^CD5^bright^CD21^−^, and CD3^−^CD5^−^CD21^−^ cells, freshly isolated PBMCs were stained with Violet Cell Trace Dye, and stimulated with ConA, NK cell-sensitive CTAC cells, IL-15, IL-15 and IL-2, or CTAC cells and IL-15 with or without IL-2 (Figure 6). CD3^+^CD5^dim^CD21^−^ lymphocytes showed proliferation rates similar to CD3^−^CD5^−^CD21^−^ cells following cytokine stimulation, NK-sensitive tumor cells, or a combination of these factors. However, CD3^+^CD5^bright^CD21^−^ cells had different proliferation rates compared to CD3^+^CD5^dim^CD21^−^ and CD3^−^CD5^−^CD21^−^ cells after stimulation (*p* < 0.001). CD3^+^CD5^dim^CD21^−^ and CD3^−^CD5^−^CD21^−^ cells proliferated actively after stimulation with IL-15, IL-15 and IL-2, CTAC cells, CTAC cells and IL-15, and CTAC cells, IL-15, and IL-2, whereas minor proliferation rates were observed for CD3^+^CD5^bright^CD21^−^ cells. Proliferation of CD3^+^CD5^bright^CD21^−^ cells (95.2 ± 0.7%) after ConA stimulation was markedly higher compared to CD3^+^CD5^dim^CD21^−^ (80.5 ± 2.8%) and CD3^−^CD5^−^CD21^−^ (77.5 ± 2.2%) cells (*p* < 0.001). Unexpectedly, CD3^−^CD5^−^CD21^−^ cells showed a similar response as CD3^+^CD5^dim^CD21^−^ cells after ConA stimulation (Figure 6).

## Discussion

Several studies on the phenotypic characteristics of canine NK cells that intended to distinguish these cells from other lymphocytes, including B and T cells, have been conducted, but no consensus on canine NK cell markers has been reached to date (14–22). Although CD3^−^CD21^−^ large granular lymphocytes that express NKp46 (NCR1) are thought to be a population of canine NK cells, it is clear that NKp46 cannot be used as a marker for all NK cells since there is a large subset of circulating NKp46^−^ NK cells that can be induced to express NKp46 in dogs (14–16). Furthermore, CD3^+^NKp46^+^ and CD5^dim^NKp46^+^ populations were also observed in PBMCs in most healthy dogs, although the phenotype and function of these populations have not been fully characterized (14–16, 32). CD3^+^NKp46^+^ lymphocytes were found to have NK cell characteristics in pigs (33) and cattle (34). In this study, NKp46 expression patterns were not investigated in both CD3^+^CD5^dim^CD21^−^ and CD3^−^CD5^−^CD21^−^ cell populations, because NKp46-specific mAbs were difficult to obtain. However, NKp46 is undoubtedly an important receptor of NK cells, and further studies should be conducted to explore the expression pattern of this molecule within CD5^+^ and CD5^−^ populations as well as in CD3^+^ and CD3^−^ populations.

Several reports demonstrated that canine NK cells share the phenotypic characteristics of T lymphocytes (19–23). We previously reported that CD3^+^CD5^dim^CD21^−^ cells, selectively expanded *ex vivo* for 14 days, exhibited similar morphologic, genetic, and functional characteristics as canine NK cells, but were not NKT cells (17). In our subsequent studies, it was confirmed that CD3^−^CD5^−^CD21^−^ NK cells were rapidly expanded following vigorous proliferation of CD3^+^CD5^dim^CD21^−^ cells by prolonging the culture time (24–26). Consistent with previous reports, CD3^+^CD5^dim^CD21^−^ cells were also selectively expanded and became dominant in the culture 10–14 days after stimulation. After about a week, a sudden increase in the CD3^−^CD5^dim^CD21^−^ and CD3^−^CD5^−^CD21^−^ cell populations and a sudden decrease in the proportion of CD3^+^CD5^dim^CD21^−^ cells were observed. CD3^−^CD5^−^CD21^−^ cell numbers then increased rapidly, and comprised the majority of cells in culture. Various numbers of CD3^−^CD5^dim^CD21^−^ cells were observed during the cell proliferation depending on the donor (Figure 1). These cells were thought to be intermediate cells in the process of phenotype change, and showed similar characteristics to CD3^−^CD5^−^CD21^−^ cells (data not shown).

Rapid changes in the phenotypes of proliferating cells observed during culture suggest that phenotype switching occurs between the two populations in response to activation. To verify this hypothesis, we determined which cell populations were proliferating during culture through intracellular staining with Ki-67 which is an indicator of cell proliferation (Figures 2A,B). CD3^+^CD5^dim^CD21^−^ cells became a major population after 10 days of culture, and accounted for up to 78% of Ki-67-expressing cells. The phenotype of Ki-67 cells then changed to CD3^−^CD5^−^CD21^−^ without increased apoptosis (Figures 2A,B,E,F), suggesting that expanded CD3^−^CD5^−^CD21^−^ cells were derived from CD3^+^CD5^dim^CD21^−^ cells by phenotypic modulation. Most Ki-67^+^ cells in both populations expressed Granzyme B (Figure 2C). The phenotypic modulation between these two populations was confirmed by culture of purified CD3^+^CD5^dim^CD21^−^ cells, and phenotyping these cells after culture (Figure 3). CD3^−^CD5^−^CD21^−^ cells were expanded from CD3^+^CD5^dim^CD21^−^ cells, and the phenotype of most cells changed to CD3^−^CD5^−^CD21^−^ or CD3^−^CD5^dim^CD21^−^ after 21 days of stimulation (Figure 3B; Figure S3 in Supplementary Material). Consistent with previous reports (17, 24), most of the CD3^+^CD5^dim^CD21^−^ cells in PBMCs before culture expressed TCRαβ (87.2 ± 2.2%) or TCRγδ (8.2 ± 5.3%). However, the number of TCR-expressing cells in CD3^+^CD5^dim^CD21^−^ and CD3^−^CD5^dim^CD21^−^ populations decreased rapidly during culture, and more than 98% of expanded CD3^−^CD5^−^CD21^−^ cells did not express TCRαβ or TCRγδ after 21 days of culture (Figures S3 and S4 in Supplementary Material). These results indicate that canine NK cells share some of the characteristics of T cells.

Natural killer and T cells may be derived from a common precursor in mice and humans, and the precursor cell undergoes differentiation toward either T or NK cell lineages depending on the microenvironment (35, 36). Several reports have shown that T cell clones undergo a genetic reprogramming of their signaling properties, resulting in conversion to functional NK cells in patients with chronic inflammatory diseases (37). In addition, NK-like programming of T cells with reduced TCR expression could be driven through chronic activation of TCR (38). Several studies have shown that T cells can be reprogrammed into NK-like cells, which are morphologically, genetically, and functionally similar to conventional NK cells, after deletion of a specific transcription factor in mice (37). This is accompanied by a concomitant loss of T cell-related gene expression, including CD3 (39). In this study, CD3^+^CD5^dim^CD21^−^ cells responded to stimulation with cytokines, NK cell-sensitive tumor cells, or a combination of these factors in a similar manner to NK cells (33) and CD3^−^CD5^−^CD21^−^ cells (Figure 6). These results indicate that a subset of NK cells other than γδ T, NKT, and CD8 cytotoxic T cells, may be contained in the CD3^+^CD5^dim^CD21^−^ population. Both CD3^+^CD5^dim^CD21^−^ and CD3^−^CD5^−^CD21^−^ cells also proliferated in response to ConA, which is a known T cell-specific mitogen (Figure 6). It has been reported that NK cells can be proliferated in cultures of PBMCs stimulated with ConA by various cytokines produced by bystander cells such as CD4^+^ T cells (40–42). However, minor proliferation after ConA stimulation was reported for NK cells in cultures of porcine PBMCs (33). Further studies are needed to determine whether proliferation of CD3^−^CD5^−^CD21^−^ cells was due to the directed effect of ConA or bystanding stimulations.

Taken together, CD3^+^CD5^dim^CD21^−^ and CD3^−^CD5^−^CD21^−^ cells both contain a subset of putative NK cells, and the difference between the two populations may be due to the degree of maturation. Further studies should be conducted to determine the precise molecular and signaling mechanisms of CD3^+^CD5^dim^CD21^−^ to CD3^−^CD5^−^CD21^−^ cell phenotypic modulation.

The T-box transcription factors T-bet and Eomes have been well defined as master regulators of NK cell development, maturation, and function in mice and humans (30, 31). In addition, the majority of mature NK cells in peripheral blood coexpress both factors for stability or development at alternative stages of maturation (30, 43). Therefore, we evaluated the expression of T-bet and Eomes in lymphocyte subsets taken from freshly isolated PBMCs, and further examined changes in the expression of both factors during culture (Figure 5). A significantly higher frequency of CD3^+^CD5^dim^CD21^−^ cells expressed T-bet and Eomes compared to CD3^−^CD5^−^CD21^−^ or CD3^+^CD5^bright^CD21^−^ cells in freshly isolated PBMCs, and the majority of CD3^+^CD5^dim^CD21^−^ cells coexpressed both factors (*p* < 0.001). The expression of these transcription factors in CD3^+^CD5^dim^CD21^−^ cells significantly decreased with time during culture (*p* < 0.05), and the expression of Eomes reached similar levels as that found in CD3^−^CD5^−^CD21^−^ cells. However, T-bet expression in CD3^+^CD5^dim^CD21^−^ cells was still higher than CD3^−^CD5^−^CD21^−^ cells after 2 weeks of culture. The expression of both factors in proliferated CD3^−^CD5^−^CD21^−^ cells after culture was not different from those in the same population of PBMCs before culture (Figure 5). The expression of T-bet and Eomes in CD3^+^CD5^dim^CD21^−^ cells from freshly isolated PBMCs was similar to peripheral blood NK cells in humans (30). However, the expression of these factors in canine NK-like cell populations undergoing phenotypic modulation was different from NK cells in both mice and humans, which upregulate T-bet and downregulate Eomes during maturation (31, 43). The downregulation of T-bet and Eomes is a molecular signature of NK cell exhaustion in mice (44), and can be induced by homeostatic proliferation in lymphopenic environments in humans (45). A previous study showed that impaired NK function caused by the downregulation of T-bet and Eomes could be overcome by IL-15 administration in mice (31, 46). These could be associated with the downregulation of the mRNA levels of several NK-related genes in CD3^−^CD5^−^CD21^−^ cells compared to CD3^+^CD5^dim^CD21^−^ cells after culture, although the cytotoxic function of CD3^−^CD5^−^CD21^−^ cells was similar to that of CD3^+^CD5^dim^CD21^−^ cells (Figures 4A,C). CD3^−^CD5^−^CD21^−^ cells were proliferated vigorously during the culture with the stimulation of cytokines including IL-15. After 21 days of culture, these cells were gradually exhausted, and were found to lose their functions (data not shown). Little is known about the function of T-bet and Eomes in NK cell biology in dogs, and further studies should be performed to identify the role of T-bet and Eomes in the transcriptional regulation of NK cell development, maturation, and function. T-bet and Eomes modulate many NK cell effector functions, including cytotoxicity and cytokine production (31, 43). In addition, we found a positive correlation between T-bet levels and mRNA levels of Granzyme B and IFN-γ production in canine NK-like cells (Figures 4 and 5; Figure S6 in Supplementary Material). However, the protein levels of Granzyme B in CD3^−^CD5^−^CD21^−^ and CD3^+^CD5^dim^CD21^−^ cells after 2 weeks of culture did not match the mRNA levels of that in the same cell population (Figures 2C,D and 4A; Table S1 in Supplementary Material). These results were thought to be due to rapid phenotype modulation from CD3^+^CD5^dim^ to CD3^−^CD5^−^, and the intermediate cells in the process of phenotypic modulation in donors with very rapid cell proliferation as shown in donor 2 of Figure 1A (CD3^+^CD5^dim^CD21^−^ population at day 10, day 14). It has been reported that the protein levels of Granzyme B did not correlate well with its mRNA (47, 48).

In summary, we provide strong evidence that putative canine NK cells can be induced from a cell subset which shares some T cell characteristics. Unlike other animal species, a subset of canine NK-like cells is contained in the CD3^+^CD5^dim^CD21^−^ population, which can differentiate into non-B, non-T NK (CD3^−^CD5^−^CD21^−^ GranzymeB^+^) lymphocytes through cytokine stimulation. *In vitro* studies of PBMCs or purified CD3^+^CD5^dim^CD21^−^ cells confirmed that proliferated CD3^−^CD5^−^CD21^−^ cells were derived from CD3^+^CD5^dim^CD21^−^ cells through phenotypic modulation. CD3^+^CD5^dim^CD21^−^ cells showed more NK cell functional characteristics than CD3^−^CD5^−^CD21^−^ cells, including the production of Granzyme B and IFN-γ, and the expression of NK cell-related molecular receptors such as NKG2D and NKp30. Overall, the results of this study suggest that both CD3^+^CD5^dim^CD21^−^ and CD3^−^CD5^−^CD21^−^ cells are putative canine NK cells, and that the difference between the two populations is the degree of maturation.

## Ethics Statement

The use of animals for this study was approved by the Institutional Animal Care and Use Committee of Kongju National University (KNU_2017-02).

## Author Contributions

S-HL, D-JS, YK, C-JK, and TU performed the experiments. S-KK, G-HS, and MY designed the experiments. J-JL, J-YJ, DY, DC, and B-GJ interpreted the data. S-KK and S-HL drafted the manuscript. All authors approved of the final manuscript for publication.

## Conflict of Interest Statement

The authors declare that the research was conducted in the absence of any commercial or financial relationships that could be construed as a potential conflict of interest.
