# Supplementary material for: Comparison of Phenotypic and Functional Characteristics Between Canine Non-B, Non-T Natural Killer Lymphocytes and CD3+CD5dimCD21− Cytotoxic Large Granular Lymphocytes
Source: Front Immunol. 2018 Apr 27;9:841. doi: 10.3389/fimmu.2018.00841 (PMC5934500; doi:10.3389/fimmu.2018.00841)
Supplement: Supplementary file 1 [file data_sheet_1.docx]

Supplementary Material

**Comparison of phenotypic and functional characteristics between canine non-B, non-T NK lymphocytes and CD3^+^CD5^dim^CD21^–^** **cytotoxic large granular lymphocytes**

Soo-Hyeon Lee, Dong-Jun Shin, Yoseop Kim, Cheol-Jung Kim, Je-Jung Lee, Mee Sun Yoon, Tung Nguyen Thanh Uong, Dohyeon Yu, Ji-Youn Jung, Duck Cho, Bock-Gie Jung, Sang-Ki Kim*, Guk-Hyun Suh*

*** Correspondence:** Sang-Ki Kim: [sangki@kongju.ac.kr](mailto:sangki@kongju.ac.kr), and Guk-Hyun Suh: ghsuh@ chonnam.ac.kr

**Supplementary Figure 1**

**
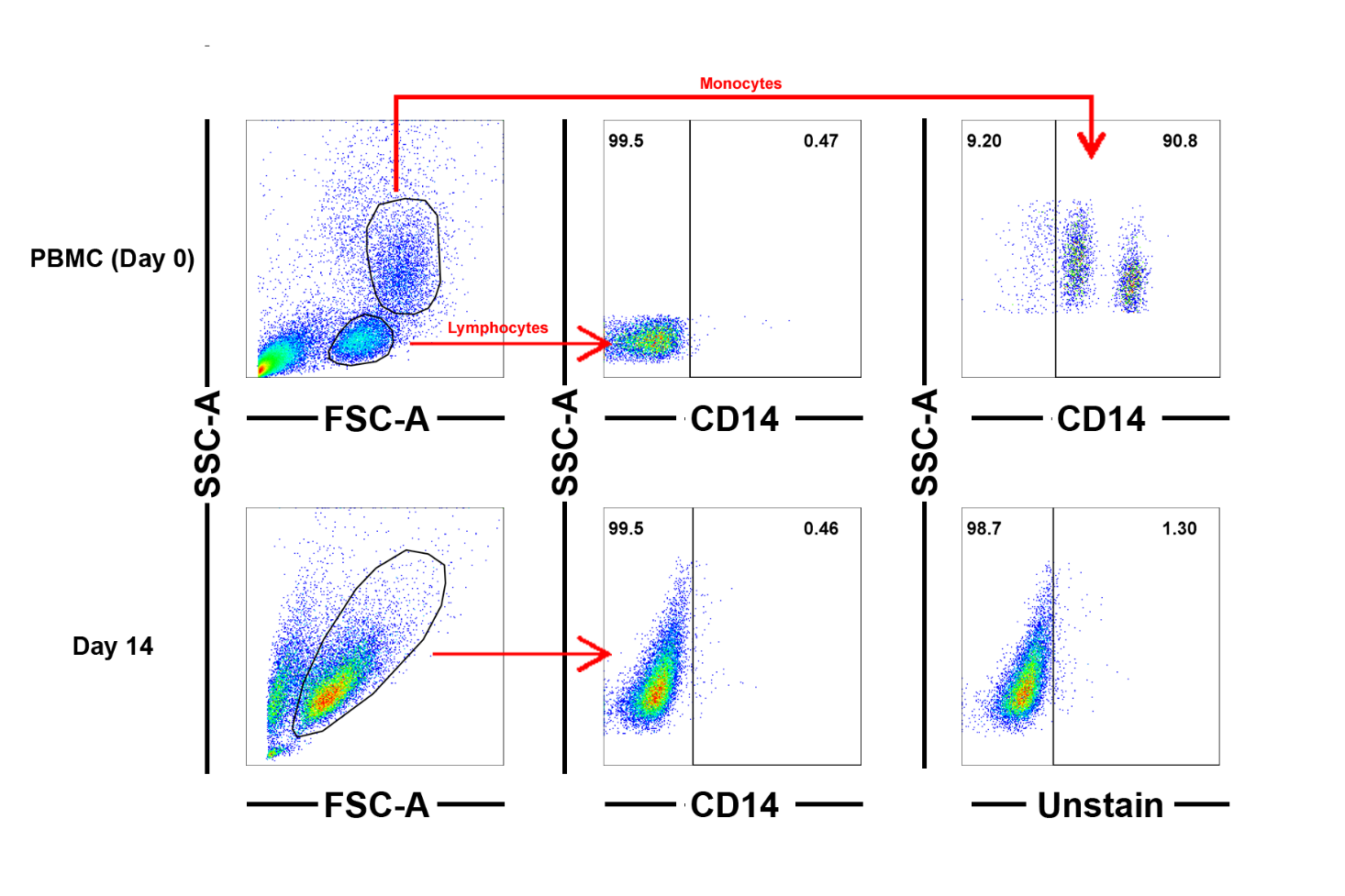
**

**Figure S1. The frequency of CD14^+^ monocytes in cells cultured for 14 days**. (A) Binding of anti-human CD14 antibody (clone M5E2) to canine monocytes in PBMCs was confirmed. (B) CD14^+^ monocytes were not present in cells cultured for 14 days.

**Supplementary Figure 2**


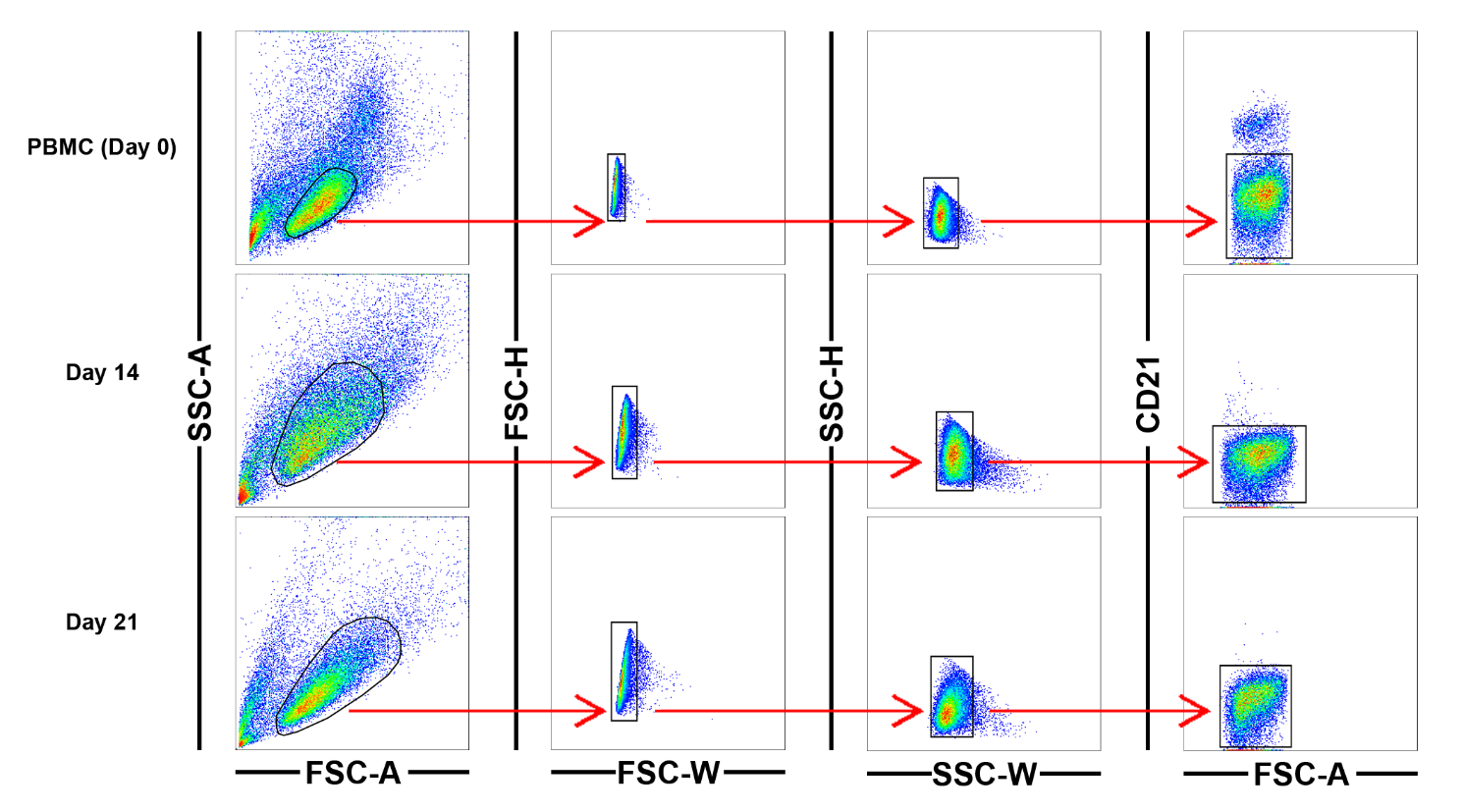


**Figure S2.** **Gating strategy for CD21 negative lymphocytes.** B cells expressing CD21 were excluded before cell purification or flow cytometry analysis of CD3^+^CD5^dim^, CD3^+^CD5^bright^, and CD3^–^CD5^–^ cells in freshly isolated PBMCs (day0) and cultured cells (day14, day21).

**Supplementary Figure 3**

**
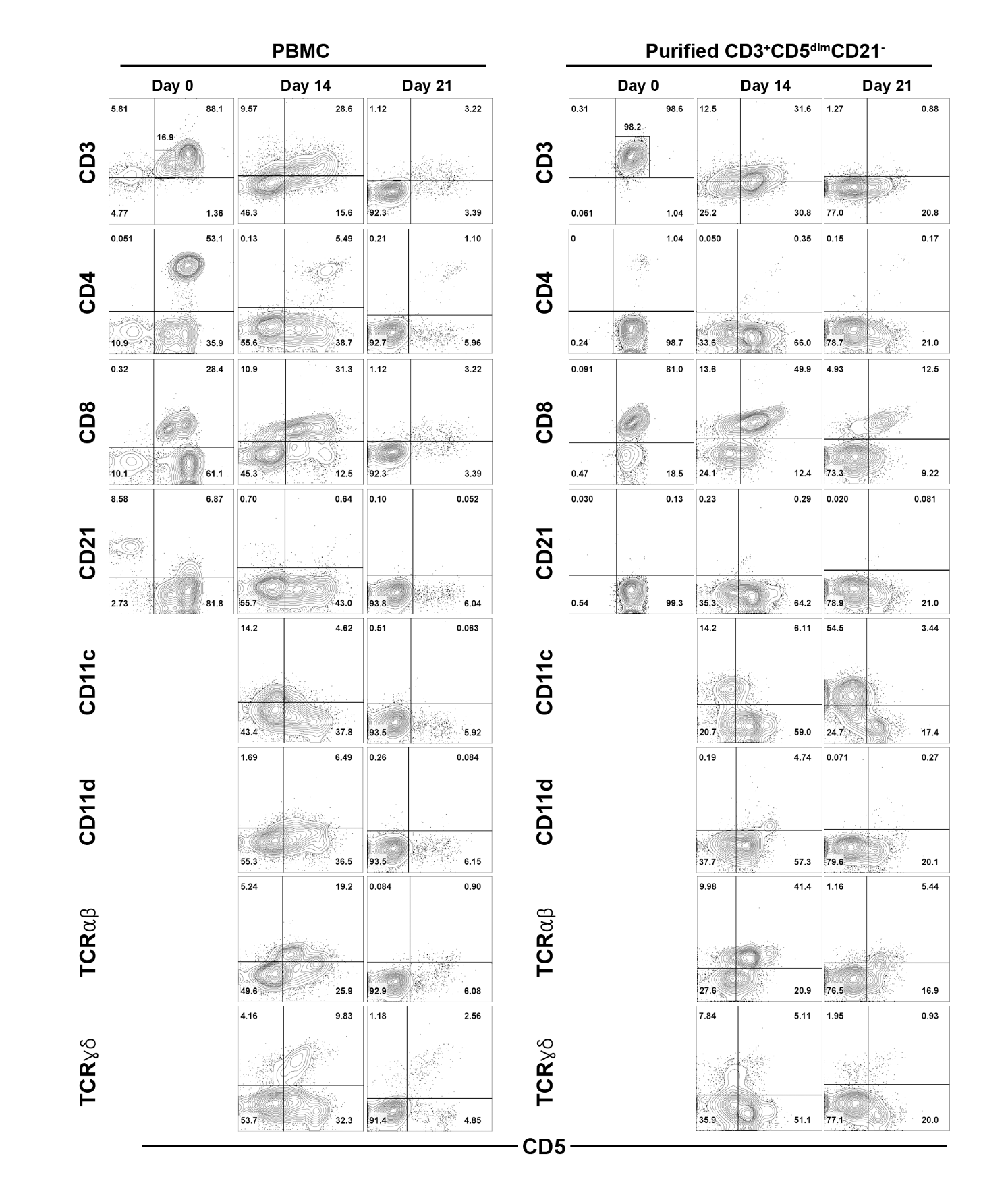
**

**Figure S3.** Phenotypic changes of PBMCs and purified CD3^+^CD5^dim^CD21^–^ cells of the same donor during culture for 21 days. Both cell populations were cultured in the same day. Cell proliferation was more rapid and vigorous in PBMCs than in purified cells.

**Supplementary Figure 4**


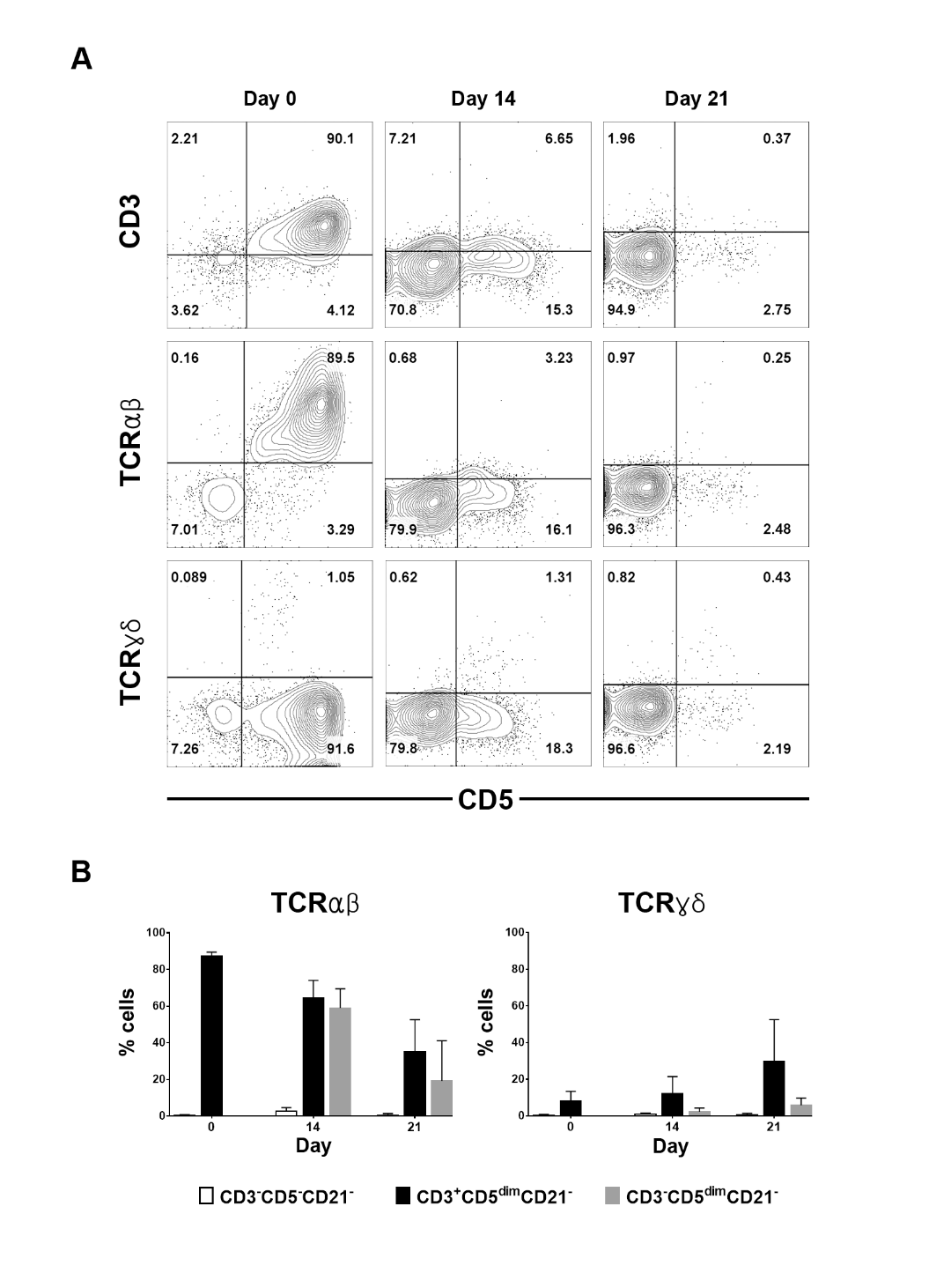


**Figure S4.** Changes in expression of TCRαβ and TCRγδ on lymphocyte subpopulations during culture of PBMCs for 21days. (A) Representative flow cytometry data (n=6). (B) Changes in the frequency of TCRαβ or TCRγδ expression on CD3^+^CD5^dim^CD21^–^, CD3^–^CD5^dim^CD21^–^, and CD3^–^CD5^–^CD21^–^ cell populations during PBMC culture for 21 days. The results are shown as the mean ± standard deviation (SD) measured from 6 different donors.

**Supplementary Figure 5**


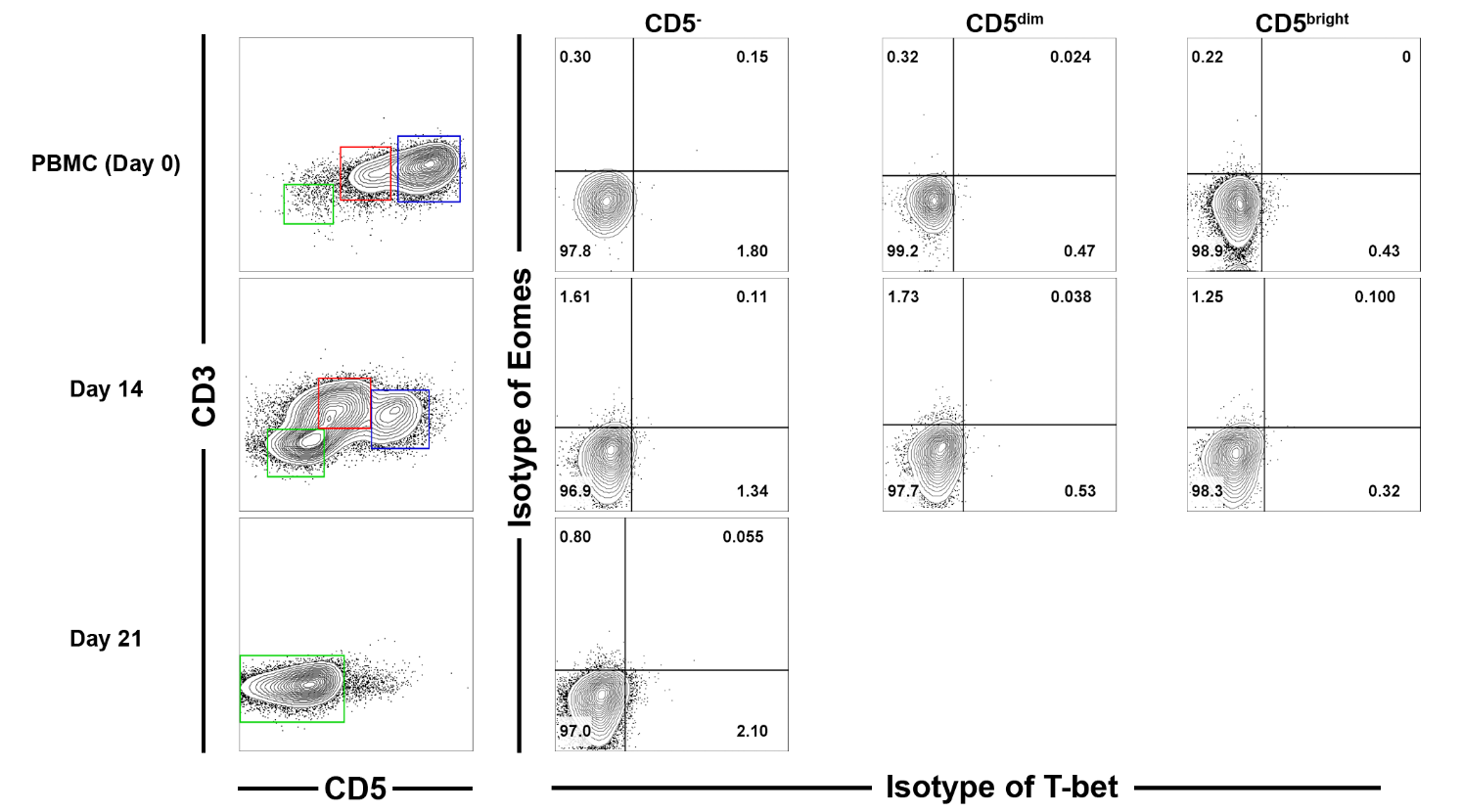


**Figure S5.** Gating strategy and fluorescence minus one (FMO) controls to evaluate the expression of T-bet and Eomes in CD3^+^CD5^dim^CD21^–^ (CD5^dim^, red), CD3^+^CD5^bright^CD21^–^ (CD5^bright^, blue), and CD3^–^CD5^–^CD21^–^ (CD5^–^, green) lymphocytes.

**Supplementary Figure 6**


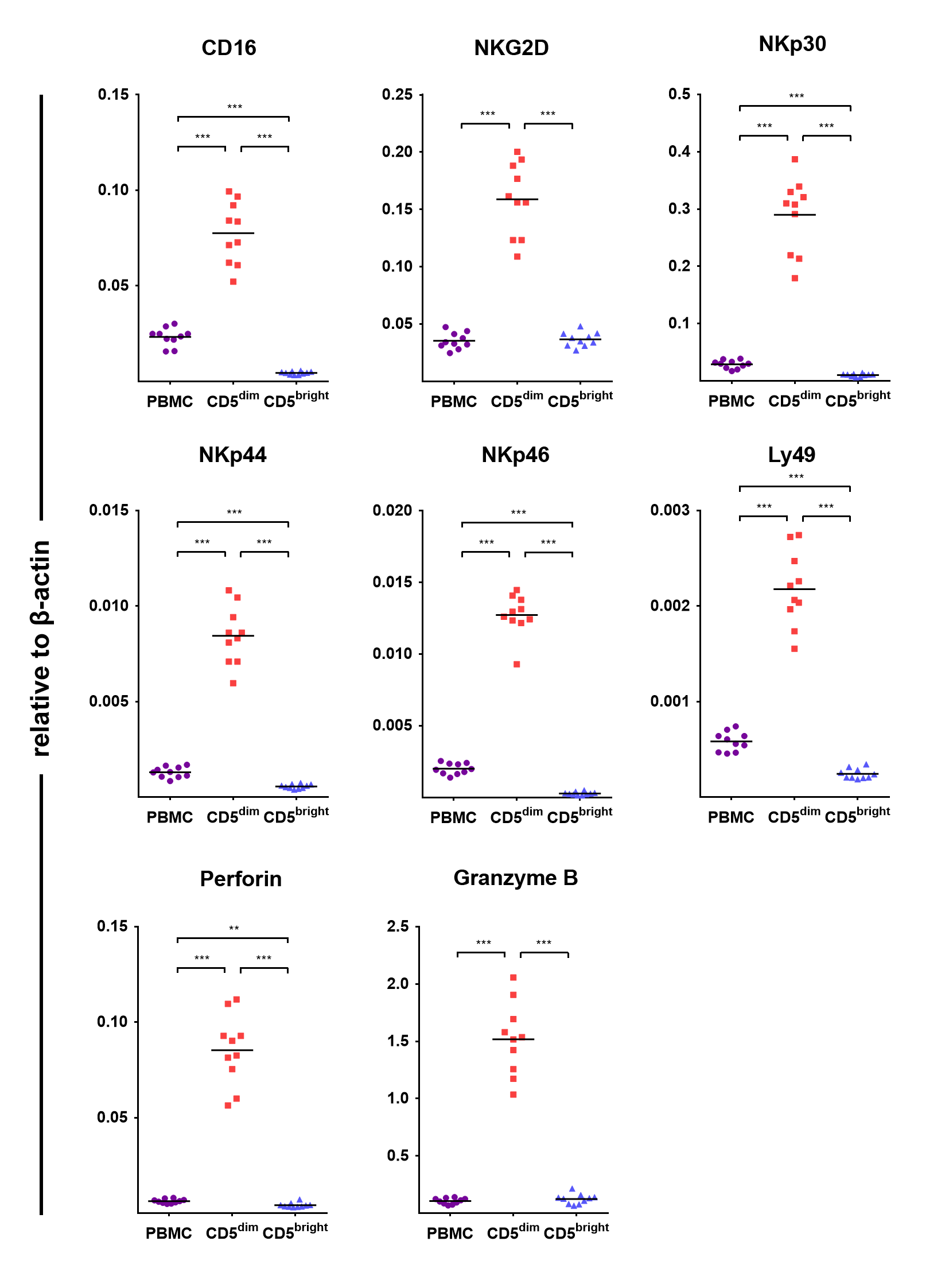


**Figure S6.** Expression of NK-associated receptor of CD3^+^CD5^dim^CD21^–^ (CD5^dim^) and CD3^+^CD5^bright^CD21^–^ (CD5^bright^) lymphocytes purified from PBMCs.

**Supplementary Table S1**

**Table S1.** Summary of the relative expression of NK-related genes, Ki-67, T-bet, and Eomes in CD3^+^CD5^dim^CD21^–^ (CD5^dim^),

CD3^+^CD5^bright^CD21^–^ (CD5^bright^), and CD3^–^CD5^–^CD21^–^ (CD5^–^) lymphocytes on day 0 (PBMCs), day 14 and day 21 of culture.

|  | PBMCs | | | | Day 14 | | | Day 21 | |
| --- | --- | --- | --- | --- | --- | --- | --- | --- | --- |
|  | **CD5^-^** | **CD5^dim^** | **CD5^bright^** | **CD5^-^** | | **CD5^dim^** | **CD^5bright^** | **CD5^-^** |  |
| Intracellular staining |  | | | | | | | | |
| Ki-67 | Low | Low | High | High | | High | Low | High |  |
| Granzyme B | Intermediate | Intermediate | Low | High | | High | High | low |  |
| T-bet^+^ | Intermediate | High | Low | Intermediate | | High | Intermediate | Intermediate |  |
| Eomes^+^ | Intermediate | High | Low | Intermediate | | Intermediate | Intermediate | Intermediate |  |
| T-bet^+^Eomes^+^ | Intermediate | High | Low | Low | | Intermediate | Low | low |  |
|  | | | | | | | | | |
| mRNA levels |  | | | | | | | | |
| CD16 | NT | High | Low | Low | | High | Low | low |  |
| NKG2D | NT | High | Low | Low | | High | Low | Intermediate |  |
| NKp30 | NT | High | Low | Low | | High | Low | low |  |
| NKp44 | NT | High | Low | High | | High | Low | high |  |
| NKp46 | NT | High | Low | Intermediate | | High | Intermediate | High |  |
| Ly49 | NT | High | Low | Low | | High | Low | low |  |
| Perforin | NT | High | Low | Intermediate | | Intermediate | Low | High |  |
| Granzyme B | NT | High | Low | Low | | High | Low | low |  |

NT, not tested.
